# The efficacy and safety of 5-fluorouracil/cisplatin/vincristine as a multi-agent chemotherapy regimen in gestational trophoblastic neoplasia

**DOI:** 10.3389/fonc.2023.1240972

**Published:** 2023-10-31

**Authors:** Lu Wang, Qian Wang, Zhen Xu, Linli Yang, Wuliang Wang

**Affiliations:** ^1^ Department of Obstetrics and Gynecology, The Second Affiliated Hospital of Zhengzhou University, Zhengzhou, China; ^2^ Henan Provincial Clinical Research Center for Gynecological and Obstetrical Disease (Gynecological Oncology), Zhengzhou, China

**Keywords:** gestational trophoblastic neoplasia, multi-agent chemotherapy, FAV, pulsed intravenous, efficacy

## Abstract

**Objective:**

To determine the efficacy and safety of the 5-fluorouracil (5-FU), cisplatin, and vincristine (FPV) chemotherapy regimen in patients with gestational trophoblastic neoplasia (GTN).

**Methods:**

We performed a retrospective study of 96 GTN patients with International Federation of Gynecology and Obstetrics (FIGO) scores of 5 or greater in the Second Affiliated Hospital of Zhengzhou University from October 2013 to October 2019, including 54 patients who received FPV chemotherapy and 42 who received 5-FU/actinomycin D/vincristine (FAV) chemotherapy. A pulsed intravenous device was used to administer 5-FU. The clinical characteristics, adverse events, and response rates were compared between the groups.

**Results:**

The patients in the FPV and FAV groups received a total of 228 and 190 courses of chemotherapy, respectively. Complete response (CR) was found in 88.89% (48/54) and 90.48% (38/42) of patients in the FPV group and FAV group, respectively (*p* = 0.801). Both chemotherapy regimens yielded CR in all low-risk patients (100% vs. 100%), whereas 86.67% and 88.24% of high-risk patients achieved CR (FPV vs. FAV, *p* = 0.836), respectively. The most common adverse events (AEs) were myelosuppression and gastrointestinal reactions including neutropenia (83.97%), anemia (60.05%), and nausea (46.41%). In comparison to those in the FAV group, patients in the FPV group reported higher rates of grade 1/2 nausea (53.51% vs. 37.89%, *p* = 0.001), hepatotoxicity (28.95% vs. 17.89%, *p* = 0.008), oral mucositis (23.25% vs. 10.53%, *p* = 0.001), and grade 3/4 neutropenia (47.37% vs. 27.37%, *p* < 0.001), while grade 1/2 diarrhea (7.46% vs. 13.68%, *p* = 0.037) and grade 3/4 oral mucositis (0 vs. 6.32%, *p* < 0.001) were much more common in the FAV group. The rate of overall survival at 5 years was 96.8% in the FPV group and 97.3% in the FAV group (*p* = 0.760), whereas the 5-year disease-free survival rates were 95.9% and 93.9% (*p* = 0.754), respectively.

**Conclusion:**

The FPV and FAV regimens with pulsed intravenous 5-FU yielded comparable CR rates and tolerability in patients with GTN with FIGO scores of >5. Further randomized controlled trials are warranted to validate their efficacy.

## Introduction

Gestational trophoblastic neoplasia (GTN) refers to the invasive and malignant forms of gestational trophoblastic disease that arises in the placental tissue and includes invasive mole, choriocarcinoma, placental-site trophoblastic tumors, and epithelioid trophoblastic tumors. Postpartum abnormal vaginal bleeding often occurs after non-molar pregnancy, whereas post-molar GTN is usually diagnosed by asymptomatic human chorionic gonadotropin (hCG) surveillance. Owing to the aggressive nature of trophoblast cells, GTN lesions can affect the myometrium, lungs, liver, and even the brain, causing bleeding from metastatic sites and neurological signs of spinal or brain metastasis. Treatment of GTN generally includes chemotherapy owing to its highly chemosensitive nature. The best regimen depends on stage and classification. In the 2000 International Federation of Gynecology and Obstetrics (FIGO) staging and classification, a risk score of 6 and below is classified as low risk and above 6 is considered high risk (1). According to the FIGO cancer report 2021, for patients with low-risk GTN, a single agent of methotrexate or actinomycin D (Act-D) would suffice as the primary treatment, and for patients with high-risk GTN, multi-agent chemotherapy regimens, such as EMA-CO (etoposide, methotrexate, Act-D, cyclophosphamide, and vincristine), are often used (2). Hysterectomy and laparotomy might be considered for uncontrolled bleeding of the uterus, liver, gastrointestinal tract, kidneys, or spleen. Radiotherapy may be helpful for treating brain metastases. Monitoring the specific biomarker of hCG together with the development of highly effective therapies has achieved a cure rate of 80%–90%.

In 2016, the Cochrane Review included 667 patients in seven randomized controlled trails and reported that in women with low-risk GTN, Act-D was more likely to achieve a primary cure than methotrexate (MTX) (3). Act-D may be associated with a greater risk of severe adverse events (SAEs) than the methotrexate regimen; however, a pulsed Act-D regimen could reduce such side effects. A meta-analysis also demonstrated that 5-day intravenous (IV) Act-D and pulsed IV Act-D appeared to be the best treatment options for low-risk GTN because of their higher complete remission (CR) rates and lower toxicity than MTX-based regimens (4). The risk of resistance to single-agent chemotherapy is increased in patients with a higher risk score of 5–6 and a clinical or pathologic diagnosis of choriocarcinoma. Thus, multi-agent chemotherapy can be considered for those patients (2). Various combinations of multi-agent chemotherapy for resistant or recurrent GTN and high-risk GTN includes EMA-CO, MEA (MTX, etoposide, and Act-D), MAC (MTX, Act-D, and chlorambucil), FA (5-FU and Act-D), FAV (5-FU, Act-D, and vincristine), MEF (MTX, etoposide, and 5-FU), EMA/EP (etoposide, MTX, Act-D/etoposide, and cisplatin), and CHAMOCA (MTX, Act-D, cyclophosphamide, doxorubicin, melphalan, hydroxyurea, and vincristine) (5). However, the optimal combination remained unclear.

Because of the high remission rate and mild toxicity of 5-FU, regimens including 5-FU are favored in China, and some regimens are effective as primary treatments for low- and high-risk GTN (2, 6, 7). The FAV regimen is a classic treatment for high-risk GTN, with satisfactory efficacy. However, owing to the relatively severe side effects of FAV and shortage of domestic Act-D, substitute regimens have been adopted. In the current study, we report the clinical efficacy and side effects of FPV (5-FU, cisplatin, and vincristine) compared to those of FAV in our center, with the aim of providing an alternative chemotherapy regimen for GTN.

## Methods

### Study population

We retrospectively included 96 patients who received FPV or FAV chemotherapy for GTN between October 2013 and October 2019. The inclusion criteria were as follows: (1) age > 18 years, (2) diagnosis of low-risk GTN (FIGO score: 5–6) or high-risk GTN, and (3) complete clinical data. Clinical data before treatment were collected, including medical history, physical examination, pelvic ultrasonography, complete blood count, hepatorenal function, serum *β*-human chorionic gonadotropin (*β*-HCG), and chest X-ray or computerized tomography (CT) scan. Brain and liver CT or magnetic resonance imaging (MRI) scans were subscribed only when positive findings were discovered on chest x-ray or CT. Serum *β*-HCG level before treatment was defined as the value of the day or 1 day before chemotherapy. Informed consent for chemotherapy was obtained before study commencement. This retrospective cohort study was approved by the Ethics Committee of the Second Affiliated Hospital of Zhengzhou University and was performed in accordance with the principles of the Declaration of Helsinki. The requirement for informed consent was waived.

### Chemotherapy regimens and management of adverse events

Pulsed IV 5-FU was administered to both (FPV and FAV) groups. The FPV regimen was administered as follows: 1–2 mg of vincristine through IV administration 12 h before 5-FU and cisplatin were administered on day 1; 23–25 mg/(kg·d) 5-FU through continuous intravenous administration, pumped by a Baxter adjustable portable infusion device; and 20 mg/(kg·d) cisplatin through an IV drip on days 2–9. The FAV regimen was administered as follows: 1–2 mg of IV vincristine 12 h before 5-FU and Act-D were administered on day 1; 23–25 mg/(kg·d) 5-FU through continuous IV, pumped by a Baxter adjustable portable infusion device; and 4–6 μg/kg IV drip of Act-D on days 2–9. The regimens were repeated every 3 weeks. Serum *β*-HCG monitoring was performed before initiation of therapy and each dose of chemotherapy. For patients with low-risk GTN, one to two extra courses of chemotherapy were administered after the serum *β*-HCG level normalized, while patients with high-risk GTN received two to three extra courses.

Adverse events (AEs) of hematologic and non-hematologic toxic reactions were recorded according to the National Cancer Institute Common Terminology Criteria for Adverse Events (version 5.0; http://ctep.cancer.gov). Before and during chemotherapy, antiemetic and gastric acid-inhibitory drugs and medicines to protect liver function and oral mucosal were administered to avoid common AEs. Granulocyte colony-stimulating factor (G-CSF) at 5 mg/kg was administered to patients with grade 1–2 neutropenia. To decrease the incidence of severe neutropenia, 6 mg of polyethylene glycol recombinant human G-CSF (rhG-CSF) was administered to patients with previous grade 3–4 neutropenia 48 h after each treatment cycle. No treatment course began unless the neutrophil count was ≥2.0×10^9^/L and the platelet count was ≥75.0×10^9^/L. Anemia was treated with iron supplements, vitamin C, or blood transfusion until the hemoglobin concentration exceeded 80 g/L. Patients were monitored every 3 weeks for response and toxicity by physical examination, hematologic and chemistry profiles, and serum *β*-HCG levels. Changes in chemotherapy regimens were administered to patients with unrecovered AEs and treatment delays of >3 weeks.

### Primary outcome measurement and follow-ups

The primary outcome was CR of serum *β*-HCG levels as a result of therapy. Secondary outcomes were drug resistance, AEs, and survival rates. CR was defined as four consecutive weekly *β*-HCG levels less than 5 mIU/mL. The decrease of *β*-HCG level to <1 log after two consecutive courses of treatment was considered no response or resistant to chemotherapy. The elevation of serum *β*-HCG level 1 month after the cessation of chemotherapy in patients with CR was considered relapse if another pregnancy was excluded. After treatment, follow-up with monthly *β*-hCG monitoring for 12 months was carried out, then every 3 months for the second year, and then every 6 months until at least 5 years. Patients were advised to use barrier contraception for 1 year. The follow-up procedure was completed in October 2020.

### Statistical analysis

All analyses were conducted using SPSS (version 24.0; SPSS, Inc., Chicago, IL, USA). Baseline characteristics and laboratory results were summarized using descriptive statistics, including percentage and means ± standard deviation (SD). For quantitative variables, a *t*-test was used to compare group differences. For categorical variables, the chi-square test or Fisher’s exact test was used for group comparisons. Kaplan–Meier and log-rank tests were used for survival analysis. The differences were considered statistically significant at *p* < 0.05.

## Results

### Study population

Among the 96 patients included in this study, 21 had failed primary chemotherapy for persistent or relapsed GTN and were transferred to our center. The patients ranged in age from 25 to 46 years (median = 36 years). Pretreatment serum *β*-hCG levels ranged from 546 to 1.35 × 10^6^ mIU/mL (median = 2.28 × 10^4^ mIU/mL). In the FPV group, 49 patients had invasive moles and 5 had choriocarcinoma, while the FAV group included 38 patients with invasive moles and 4 with choriocarcinoma. The FIGO stages of all patients were as follows: 15 (15.63%) had stage I disease, 9 (9.38%) had stage II, 68 (70.83%) had stage III, and 4 (4.17%) had stage IV disease. With respect to the World Health Organization (WHO) prognostic scores, 17 (17.71%) patients were low risk and 79 (82.29%) were high risk. Of all the patients, 54 received FPV chemotherapy and 42 received FAV chemotherapy. The characteristics of patients with GTN at baseline were compared between the two groups and no differences in age, antecedent pregnancy, interval, pretreatment *β*-hCG level, previous chemotherapy, FIGO stage, and prognostic score were found (*p* > 0.05) (**Table 1**).

**Table 1:** Characteristics of GTN patients at baseline (*n* = 96).

	FPV group (*n* = 54, %)	FAV group (*n* = 42, %)	*p*-value
Median age (range)	38 (25–46)	35 (27–44)	0.317
<40 years	41 (75.93)	28 (66.67)	
≥40 years	13 (24.07)	14 (33.33)	
Antecedent pregnancy			0.222
Hydatidiform mole	33 (61.11)	26 (61.90)	
Abortion	8 (14.81)	2 (4.76)	
Term pregnancy	13 (24.07)	14 (33.33)	
Interval			0.084
<4 months	32 (59.26)	21 (50.00)	
4–6 months	11 (20.37)	7 (16.67)	
7–12 months	5 (9.26)	12 (28.57)	
>12 months	6 (11.11)	2 (4.76)	
Pretreatment *β*-hCG level			0.745
<1,000	4 (7.41)	5 (11.90)	
1,000–<10,000	16 (29.63)	12 (28.57)	
10,000–<100,000	21 (38.89)	18 (42.86)	
>100,000	13 (24.07)	7 (16.67)	
Previous chemotherapy			0.807
No	41 (75.93)	34 (80.95)	
Mono-chemotherapy	9 (16.67)	6 (14.29)	
Multi-agent chemotherapy	4 (7.41)	2 (4.76)	
FIGO stage			0.922
I	9 (16.67)	6 (14.29)	
II	6 (11.11)	3 (7.14)	
III	37 (68.52)	31 (73.81)	
IV	2 (3.70)	2 (4.76)	
Prognostic score			0.762
Low risk	9 (16.67)	8 (19.05)	
High risk	45 (83.33)	34 (80.95)	

*GTN, gestational trophoblastic neoplasia; FPV, 5-Fluorouracil, cisplatin, vincristine; FAV, 5-Fluorouracil, actinomycin D, vincristine; FIGO, International Federation of Gynecology and Obstetrics.

### Response to chemotherapy

**Figure 1:**
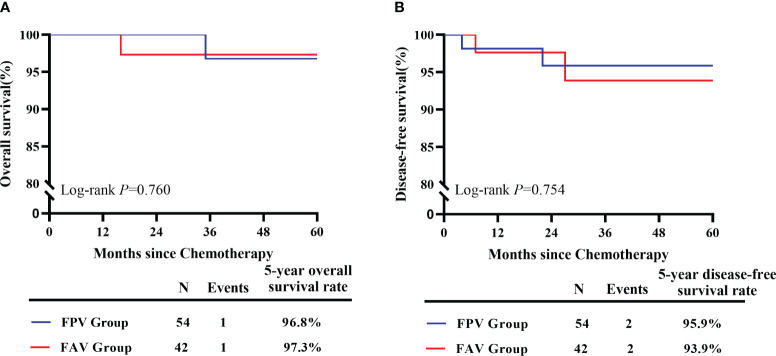
Survival estimates of included GTN patients. **(A)** The 5-year overall survival of GTN patients received FPV regimens (*n* = 54) and FAV regimens (*n* = 42). **(B)** The 5-year disease-free survival of GTN patients received FPV regimens (*n* = 54) and FAV regimens (*n* = 42).

Patients in the FPV group received 228 cycles of chemotherapy and those in the FAV group received 190 cycles in total. The median duration of chemotherapy cycles was 4 (3–8) cycles in all patients, and none of the patients abandoned chemotherapy during the initial treatment. After treatment, 86 patients (89.58%) achieved CR, and 10 patients received salvage treatment, including 6 in the FPV group and 4 in the FAV group. Two of the six patients in the FPV group had unsatisfied decreases of *β*-hCG levels and received two salvage cycles of EMA-CO chemotherapy. Two patients did not tolerate the AEs of FPV and switched to the EMA-CO regimen. One patient had an elevated *β*-HCG level before it decreased to normal, and received an emergency hysterectomy for uncontrolled uterine perforation and bleeding during the third interval of chemotherapy, followed by salvage EMA-CO chemotherapy. One patient required hysterectomy after treatment for personal reasons. One of the four patients in the FAV group experienced relapse after five courses of chemotherapy and received two courses of the 5-FU/Act-D/etoposide/vincristine (FAEV) regimen. The *β*-hCG showed a decrease but was still above normal level. The patient refused to continue chemotherapy and died 9 months later. Two patients had persistent low *β*-HCG level after chemotherapy and received one course of salvage Act-D/etoposide regimen and one course of consolidation chemotherapy. One patient underwent hysterectomy after treatment. The FPV regimen showed equivalent efficacy in the rate of CR in all patients, low-risk patients, and high-risk patients compared to the FAV regimen (*p* > 0.05) (**Table 2**). The median follow-up was 42.0 months (ranging from 8 to 83 months). One patient in the FPV group experienced recurrence 22 months after initial treatment and died of multiple metastasis 13 months later. One more patient in the FAV group had a recurrence at 27 months and received salvage treatment but lost follow-up at 45 months. The rate of overall survival at 5 years was 96.8% and 97.3% in the FPV and FAV groups, respectively (*p* = 0.760). The rate of disease-free survival at 5 years was 95.9% and 93.9% in the FPV and FAV groups, respectively (*p* = 0.754) (**Figure 1**).

**Table 2:** Efficacy of FPV and FAV chemotherapy in GTN patients (*n* = 96).

	FPV group (*n* = 54, %)	FAV group (*n* = 42, %)	*p*-value
Course of chemotherapy, median (range)	4 (3–8)	4 (3–8)	0.970
Additional treatment	6 (11.11)	4 (9.52)	0.801
Salvage chemotherapy	4 (7.41)	3 (7.14)	1.000
Hysterectomy	2 (3.70)	1 (2.38)	1.000
Relapse	0 (0)	1 (2.38)	1.000
Response of all patients			0.801
CR	48 (88.89)	38 (90.48)	
No response or resistant to chemotherapy	6 (11.11)	4 (9.52)	
CR of low-risk patients	9 (9/9, 100)	8 (8/8, 100)	1.000
CR of high-risk patients	39 (39/45, 86.67)	30 (30/34, 88.24)	0.836

*FPV, 5-Fluorouracil, cisplatin, vincristine; FAV, 5-Fluorouracil, actinomycin D, vincristine; GTN, gestational trophoblastic neoplasia; CR, complete remission.

### Adverse events of chemotherapy

Data regarding AEs were collected during the course of chemotherapy. No death occurred during the initial treatment. Myelosuppression and gastrointestinal reactions were the most common AEs. Of the 418 courses of chemotherapy, 351 (83.97%) reported neutropenia, 251 (60.05%) reported anemia, 194 (46.41%) reported nausea, 100 (23.92%) reported hepatotoxicity, 85 (20.33%) reported oral mucositis, 45 (10.77%) reported diarrhea, and 24 (8.13%) reported thrombocytopenia. All grade 1/2 AEs recovered after appropriate treatment, and no chemotherapy delay occurred. Grade 3/4 neutropenia was the most common severe AE, accounting for 45.58% (160/351) of all reported neutropenia. Grade 3/4 oral mucositis and diarrhea were observed in 2.87% (12/418) and 0.48% (2/418) of courses, respectively. All patients with grade 3/4 AEs recovered after appropriate treatment, and no chemotherapy delay was observed over 3 weeks. Patients in the FPV group reported higher rates of grade 1/2 nausea, hepatotoxicity, oral mucositis, and grade 3/4 neutropenia than those in the FAV group, whereas grade 1/2 diarrhea and grade 3/4 oral mucositis were more common in the FAV group (*p* < 0.05) (**Table 3**). The FPV regimen showed tolerable AE compared to the FAV regimen.

**Table 3:** Adverse events of the FPV and FAV chemotherapy (*n* = 418).

	FPV group (*n* = 228, %)	FAV group (*n* = 190, %)	*p*-value
Nausea	122 (53.51)	72 (37.89)	0.001
Grade 1/2	122 (53.51)	72 (37.89)	0.001
Grade 3/4	0 (0)	0 (0)	–
Diarrhea	17 (7.46)	28 (14.74)	0.017
Grade 1/2	17 (7.46)	26 (13.68)	0.037
Grade 3/4	0 (0)	2 (1.05)	0.206
Oral mucositis	53 (23.25)	32 (16.84)	0.105
Grade 1/2	53 (23.25)	20 (10.53)	0.001
Grade 3/4	0 (0)	12 (6.32)	< 0.001
Neutropenia	209 (91.67)	142 (74.74)	< 0.001
Grade 1/2	101 (44.30)	90 (47.37)	0.503
Grade 3/4	108 (47.37)	52 (27.37)	< 0.001
Thrombocytopenia	25 (10.96)	9 (4.74)	0.020
Grade 1/2	22 (9.65)	8 (4.21)	0.032
Grade 3/4	3 (1.32)	1 (0.53)	0.629
Anemia	143 (62.72)	108 (56.84)	0.222
Grade 1/2	142 (62.28)	106 (55.79)	0.179
Grade 3/4	1 (0.44)	2 (1.05)	0.593
Hepatotoxicity	66 (28.95)	34 (17.89)	0.008
Grade 1/2	66 (28.95)	34 (17.89)	0.008
Grade 3/4	0 (0)	0 (0)	–

*FPV, 5-Fluorouracil, cisplatin, vincristine; FAV, 5-Fluorouracil, actinomycin D, vincristine; GTN, gestational trophoblastic neoplasia.

## Discussion

A retrospective study spanning 30 years in China revealed that patients with GTN have achieved a satisfying CR rate of 98.4% after normative initial treatment in the last 15 years (8). Low-risk GTN is a highly chemosensitive disease with a cure rate approaching 100% (9). However, a higher FIGO score of 5–6, a pathologic diagnosis of choriocarcinoma, and pretreatment hCG level > 10^5^ mIU/mL have been reported to be associated with increased resistance to first-line MTX chemotherapy (10). Thus, multi-agent chemotherapy is often administered to patients with low-risk (FIGO score: 5–6) and high-risk GTN (2). The FAV regimen is the classic regimen adopted at our center. Yet, now, because of the instability of Act-D supply, alternative solutions have been identified. In this study, we report the efficacy and safety of FPV as a multi-agent chemotherapy regimen for the treatment of GTN in a real-world setting. Our results indicated that the FPV regimen was effective and well-tolerated in patients with GTN with a FIGO score > 5.

Vincristine plays an anti-cancer role by preventing the cell from separating its chromosomes during metaphase and by inhibiting RNA synthesis (11). Act-D binds to DNA and causes DNA damage, growth inhibition, and cell death. In terms of mechanism, cisplatin is a non-specific agent of cell cycle that could act as a DNA damaging agent to kill the fastest proliferating cells (12). Cisplatin combined with paclitaxel as TP or combined with etoposide, MTX, and Act-D/etoposide as EMA/EP are the most commonly used salvage regimens for patients with resistance to EMA-CO (2, 13). 5-FU primarily inhibits thymidylate synthase, which blocks thymidine formation required for DNA synthesis and acts as an antimetabolite to prevent cell proliferation (14). Owing to its relatively short half-life (<30 min), we generally adopt an adjustable portable infusion device for pulsed intravenous 5-FU. The device can maintain a stable blood concentration of 5-FU to continuously kill tumor cells that proliferate into the S phase and does not affect the normal life of patients. Cisplatin showed a synergistic effect with 5-FU by inhibiting the transportation of methionine through cell membrane and increasing the accumulation of 5,10-methylene tetrahydrofolate, which could form a ternary complex with thymidylate synthase and 5-fluoro-2-deoxyuridylic acid monophosphate to inhibit the activity of thymidylate synthase (15). Thus, in the current study, we replace Act-D with cisplatin, and conducted a retrospective study to verify the feasibility of FPV as a substitute regimen for GTN treatment.

CR was observed in 89.58% (86/96) of the patients, despite treatment failure with previous chemotherapy. Respectively, 88.89% (48/54) of patients in the FPV group and 90.48% (38/42) in the FAV group achieved CR, which was comparable. The total CR rate was similar to that previously reported for other fluorouracil-based combined chemotherapy regimens. Li et al. investigated the efficacy of chemotherapy based on 5-FU regimen (FAV and FA) as the initial chemotherapy regimen for the treatment of high-risk GTN and found a CR rate of 87.4% (16). A retrospective study of FAEV regimen in the first-line chemotherapy of patients with GTN who had FIGO scores ≥5 reported a CR rate of 85.3% (168/207) and a 5-year overall survival rate of 97.4% (17). Furthermore, the differences in the 5-year overall survival and disease-free survival between the two groups were not statistically significant (*p* = 0.760 and *p* = 0.754, respectively). In the current study, the efficacy of the FPV regimen was similar to that of the FAV regimen in patients with low-risk GTN (FIGO scores: 5–6) and high-risk GTN. The dominant AEs were myelosuppression and gastrointestinal reactions, including neutropenia (83.97%), anemia (60.05%), and nausea (46.41%). Grade 3/4 neutropenia accounted for 45.58% of all reported neutropenia and 38.28% of all cycles. This finding is consistent with those of the previous studies. In a retrospective study of Li et al., grade 3–4 neutropenia occurred in 28.4% of cycles (17). Fortunately, all grade 3/4 AEs successfully recovered with supportive treatment, and no chemotherapy delay over 3 weeks or deaths occurred. Gastrointestinal reactions are often observed in patients undergoing chemotherapy, with nausea being the most common. The most commonly documented nausea was grade 1/2, and the FPV regimen showed a higher rate of grade 1/2 nausea than the FAV regimen (53.51% vs. 37.89%, *p* = 0.001). However, we reported no grade 3/4 nausea, which is a positive finding considering the rate of 3/4 nausea reported in previous literature. Feng et al. reported 59.3% nausea and 1.1% grade 3 nausea (18). For other types of AEs, in comparison to the FAV group, patients in the FPV group reported higher rates of grade 1/2 hepatotoxicity (28.95% vs. 17.89%, *p* = 0.008) and oral mucositis (23.25% vs. 10.53%, *p* = 0.001), whereas grade 1/2 diarrhea (7.46% vs. 13.68%, *p* = 0.037) and grade 3/4 oral mucositis (0 vs. 6.32%, *p* < 0.001) were more common in the FAV group. As reported previously, the major toxicities associated with Act-D included mucositis and alopecia (19). This may explain why there were more cases of grade 3/4 oral mucositis in the FAV group. Fortunately, all oral mucositis was alleviated following treatment with a chlorhexidine gargle or the gargle in combination with antibacterial drugs. Therefore, the toxic effects of the current FPV regimen were predictable and manageable.

The current study confirmed that the FPV regimen was an effective multi-agent regimen for patients with GTN whose FIGO scores were >5. However, continuous intravenous pumped 5-FU depending on patients’ weight may not guarantee the best curative effect. Kaldate et al. found, in a study of colorectal cancer, that proper 5-FU dosage could only be seen in 20%–30% of patients, and dose adjustment based on pharmacokinetics to ensure that area under the curve (AUC) in the desired range of 20–30 mg·h/L was recommended (20). However, Esin et al. concluded that increased plasma levels or pharmacokinetically adjusted doses of 5-FU were not related to better efficacy but increased toxicity (21). We did not adopt a pharmacokinetic dose monitor for blood concentration and could not determine the optimal concentration for drug administration. Moreover, we did not administer PD-1/PD-L1 inhibitors to patients with relapse, despite them being an alternative strategy for the management of chemoresistant or refractory GTN (22).

In conclusion, the FPV and FAV regimens with pulsed intravenous 5-FU yielded comparable CR rates and tolerability in patients with GTN with a FIGO score of >5. Gastrointestinal reactions and myelosuppression were the most common adverse events, but pulsed intravenous 5-FU was tolerable. Future multi-institutional randomized trials are warranted to determine the best chemotherapeutic choice for patients with FIGO scores >5 or chemoresistant and relapsed GTN.

## Data availability statement

The raw data supporting the conclusions of this article will be made available by the authors, without undue reservation.

## Ethics statement

The studies involving humans were approved by the Ethics Committee of the Second Affiliated Hospital of Zhengzhou University. The studies were conducted in accordance with the local legislation and institutional requirements. The participants provided their written informed consent to participate in this study.

## Author contributions

LW: Funding recipient and major contributor in writing the manuscript and analyzed the participant data. QW: Contributed to writing the manuscript and data collection. ZX: Major contributor in study design and reviewing the manuscript. LY and WW: Contributed to data analysis and management. All authors contributed to the article and approved the submitted version.
